# Starting up the community-based injury reporting system: a media for client voice 

**Published:** 2019-08

**Authors:** Homayoun Sadeghi-Bazargani, Mina Golestani, Saber Azami-Aghdash, Bahram Samadirad, Mohammad Saadati

**Affiliations:** ^*a*^Road Traffic Injury Research Center, Statistics and Epidemiology Department, Tabriz University of Medical Sciences, Tabriz, Iran.; ^*b*^Road Traffic Injury Research Center, Tabriz University of Medical Sciences, Tabriz, Iran.; ^*c*^Road Traffic Injury Research Center, Iranian Center of Excellence in Health Management, School of Management and Medical Informatics, Tabriz University of Medical Sciences, Tabriz, Iran.; ^*d*^Legal Medicine Research Center, Legal Medicine Organization, Tabriz, Iran.

## Abstract

**Background:**

Estimates show that injuries account for 9% of all deaths and the sixth leading cause of death worldwide as well as one of the major public health concerns, especially in low and middle-income countries. Given the importance of gathering relevant information on safety and injury, setting up a voluntary platform system to obtain safety reports in the pilot area, by providing appropriate analysis and feedback to stakeholders and organizations. That causes sensitivity in the country's authorities.

**Methods:**

Through systematic review Initially, basic information was extracted using scientific literature searches and reviews of successful reporting systems in other countries. The systematic review was based on the methodology introduced by CRD and PRISMA.

B) Holding expert panel meetings

After reviewing the literature from evidence and experiences in the world, several expert panel consists of 15 participants was held. Subjects were selected purpose-based to summarize and design the community-based forms of reporting for safety and injury.

C) Coordination for designing of web-based safety and injury reporting system

After initial designing the structure of the community-based safety and injury reporting system through reviewing the literatures and holding expert panel meetings, the web-based software was designed.

**Results:**

After designing the Safety and Injury Reporting platform the system was placed on the university server. The system was designed in two parts (lay people) and expert people. Then all received reports were collected and categorized by subject and type of report and then will be made available to relevant organizations to take action and follow-up the unsafe cases and urban problems. Evaluation phase will launch after receiving reports from people. It will be collected by using a semi-structured questionnaire designed by the researcher. The coefficients of CVR content validity ratio and CVI content validity index calculated.

**Conclusions:**

Voluntary reporting systems receive reports either from individuals or from organizations that have the benefit of receiving reports from individuals providing input information to physicians or researchers. Setting up a voluntary reporting system to obtain safety reports can gathering relevant information in this area, by providing appropriate analysis and feedback to stakeholders and organizations and making them sensitive to community safety.

**Keywords:**

Community-based, Injury, Reporting System, Client Voice

